# Major Royal Jelly Proteins promote C2C12 myotubes differentiation by improving mitochondrial function

**DOI:** 10.3389/fnut.2025.1636751

**Published:** 2026-01-05

**Authors:** Xin Zhang, Jinting Wei, Suchan Liao, Lingling Huang, Zhikun Bai, Yue Hu, Caiyan Yang, Bin Zhong, Yishen Gu, Biao Li, Jinhua Wang

**Affiliations:** 1 School of Basic Medical Sciences, Youjiang Medical University for Nationalities, Baise, China; 2 Affiliated Hospital, Youjiang Medical University for Nationalities, Baise, China; 3 School of Pharmacy, Youjiang Medical University for Nationalities, Baise, China; 4 Guangxi Database Construction and Application Engineering Research Center for Intracorporal Pharmacochemistry of TCM, Baise, China; 5 Moderm Industrial College of Biomedicine and Great Health, Youjiang Medical University for Nationalities, Baise, Guangxi, China

**Keywords:** Major Royal Jelly Proteins, C2C12, myoblasts, differentiation, mitochondria

## Abstract

Royal Jelly (RJ) has been widely used as a health-promoting supplement and its bioactive component is the Major Royal Jelly Proteins (MRJPs). Whether MRJPs promote skeletal-muscle-cell development remains unresolved. Muscle dysfunction is linked to mitochondrial depletion and protein breakdown. Thus, we evaluated how MRJPs affect myotube differentiation. Myotubes morphology and number were measured using fluorescence microscopy and Coomassie brilliant blue staining, and C2C12 myotube differentiation was assessed using Western blotting or qRT-PCR analysis of the expression of *MyoD*, *MyoG*, Myosin Heavy Chain (*MyHC*) and muscle ring finger 1 (*MuRF-1*). Mitochondrial function was assessed with fluorescent probes, whereas the content of mitochondria was determined by analyzing the expression of related proteins. Western blotting was used to examine the expression of myosin-associated proteins, autophagy-associated proteins, apoptosis-associated proteins, and mitochondria-associated proteins. This was demonstrated by increased myotubes density and length, and increased mRNA and proteins expression of MyoD, MyoG and MyHC. In this study, we found that MRJPs promoted the differentiation of C2C12 myoblasts to form myotubes, but could not reverse Dex-induced muscle atrophy. The possible mechanism is that MRJPs reduced apoptosis increased cellular autophagy, stimulated mitochondrial biogenesis, promoted mitochondrial dynamics homeostasis and mitophagy, and prevented the loss of mitochondrial membrane potential.

## Graphical abstract

**Figure:**
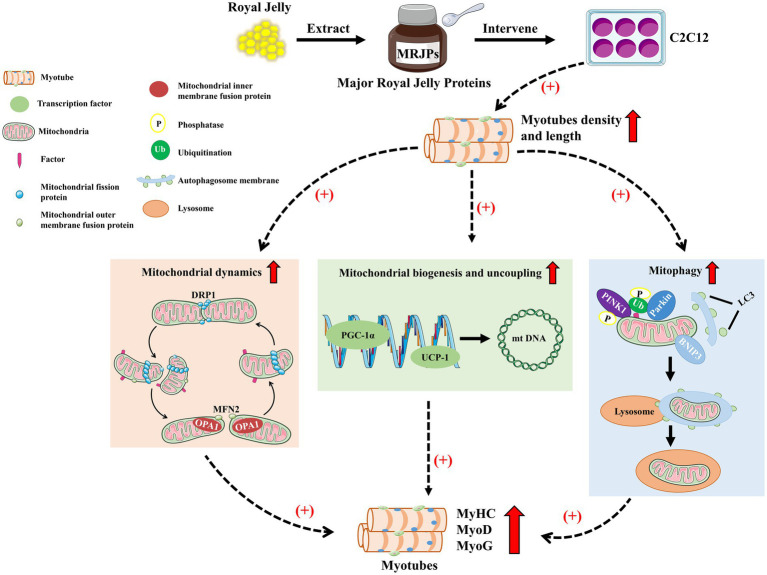
Schematic representation of the molecular mechanism of MRJPs in the induced differentiation of C2C12 myotubes. Some of the pieces in the figure were created with Servier Medical Art, and some were created with PowerPoint.

## 1 Introduction

Skeletal muscle is an important organ for regulating bodily mobility and energy levels (1). Myosatellite cells, which are located between the myosin membrane and the basal lamina of muscle fibers, play a key role in the regeneration of skeletal muscle (2). After skeletal muscle injury or degeneration, myosatellite cells are stimulated to re-enter the cell cycle and initiate the healing process in the affected area (3). Sarcopenia is a progressive and widespread skeletal muscle disease that affects older adults through an accelerated loss of muscle mass and function (4). Sarcopenia is a major health concern in older adults, increasing the risk of disability, falls and fall-related injuries, hospitalization, loss of independence, and mortality, and mortality (5). Multiple studies have shown that sarcopenia is now a growing public health problem of global concern (6).

C2C12 myoblasts are a commonly used *in vitro* model for studying skeletal muscle (7), the largest organ rich in mitochondria, which plays a vital role in activity, heat generation, and the maintenance of overall energy balance (8). Differentiation of C2C12 myoblasts is gradual, and their differentiation capacity begins to decline by day 6. Muscle bioenergetics heavily rely on mitochondrial metabolism and homeostasis, and the aging process is commonly associated with compromised mitochondrial function (9, 10). Stabilization of mitochondrial membrane potential (MMP) supports normal mitochondrial physiology. Mitochondria are major producers of reactive oxygen species (ROS). An increase in ROS leads to impaired mitochondrial function and ultimately to aging. Several natural plant extracts—including hydroxytyrosol acetate, resveratrol, and quercetin—can target mitochondria and enhance their function (11). Therefore, the development of drugs or nutrients that improve mitochondrial function may contribute to better muscle development.

Natural compounds and muscle mitochondria: Several phytochemicals enhance mitochondrial function in skeletal muscle. For example, mulberry-leaf flavonoids improve mitochondrial activity in L6 myotubes by activating the AMPK/PGC-1α axis. Likewise, the cereal-derived antioxidant betaine stimulates myogenesis and mitochondrial biogenesis in C2C12 cells (12). Royal Jelly (RJ) is a white to yellowish secretion produced by the mandibular and hypopharyngeal glands of the honeybee and contains a large number of biologically active substances (13). It is widely accepted that RJ dictates the developmental fate of female larvae. Exclusive feeding of larvae on RJ induces their maturation into queens, whose average lifespan of 1–2 years greatly exceeds that of worker bees (14). RJ has become a popular traditional health supplement, inspired by its striking effects on queen-bee longevity. Studies report that RJ exhibits anti-aging (15), anti-fatigue (16), and anti-tumor (17).

Approximately 50% of the dry weight of RJ consists of proteins (18), with 80%–90% of these proteins being Major Royal Jelly Proteins (MRJPs) (19). Whereas earlier work centred on whole royal jelly or crude extracts, we specifically interrogate the capacity of MRJPs to drive myogenesis and enhance mitochondrial function. MRJPs usually exist as oligomeric structures, and have excellent biological properties. Ten major proteins in the MRJPs family—designated MRJP1 through MRJP10—have been identified (20, 21). Xin et al. suggest that MRJPs are an important component of RJ’s ability to extend Drosophila lifespan (22). MRJPs also stimulate DNA synthesis and increases albumin production in hepatocytes, and protects cells from apoptosis through the action of several important intracellular signaling factors (23). MRJPs isolated from RJ stimulate the proliferation of various cell types, including the HFL-I cell line (24), human myeloid U-937 cells (25), human monocytes (26) and Tn-5B1-4 insect cells (27). Studies have shown that the bioavailability of MRJPs freeze-dried powder is positively affected by its processing technology, and the stability and bioavailability of its bioactive substances can be significantly improved through vacuum freeze-drying technology and other protective measures (28). Owing to inter-individual variability, the efficiency of MRJPs absorption and digestion may differ.

Nevertheless, the impact of MRJPs on the differentiation of skeletal myoblasts and the underlying mechanisms remain unclear. Thus, this work aimed to examine the impact of MRJPs on differentiation, apoptosis, autophagy, mitochondrial function, biogenesis, dynamics homeostasis, and mitophagy in C2C12 myoblasts.

## 2 Materials and methods

### 2.1 Extraction of MRJPs

As mentioned earlier, MRJPs were extracted from RJ with some modifications (29). Fresh RJ purchased from Shanghai Bee Forest Co., Ltd. was accurately weighed. It was then mixed with phosphate buffer (pH 7.0) at a 1:7 (w/v) ratio. After mixing for 4 h, the mixture was centrifuged at 12,000 rpm for 30 min at 4 °C. The supernatant was collected and the precipitate was discarded. The supernatant is then loaded into a dialysis membrane (Solarbio, YA1072) and placed in a dialyzer containing 100 volumes of ddH₂O relative to the sample volume. Finally, the dialyzer is set at 4 °C for 24 h. The dialysate was replaced after 2, 4, 6, and 12 h (total 24 h). At the end of dialysis, the sample was centrifuged at 12,000 rpm for 30 min at 4 °C. The precipitate is discarded and the supernatant was collected and lyophilized for 48 h (Alpha 1–4 LD plus, Christ, Germany) to obtain MRJP powder. The lyophilized powder is mixed with phosphate buffer solution and stored at −20 °C until assay time.

### 2.2 Measurement of total protein and MRJPs concentration

The total protein concentration was quantified using the bicinchoninic acid (BCA) protein assay kit according to the manufacturer’s instructions (Beyotime, P0010).

### 2.3 SDS-PAGE

The BCA results showed that the addition of SDS-PAGE sample buffer ensured equal protein concentrations of all the samples were the same. The samples were then boiled at 100 °C for 10 min. Samples were resolved on 4%–12% Bis-Tris polyacrylamide gels run at 80 V for 30 min followed by 120 V for 1 h. After the electrophoresis, Coomassie Brilliant Blue Rapid Staining Solution (Beyotime, P0017) was used to stain the gel.

### 2.4 C2C12 cell culture and differentiation

C2C12 mouse myoblasts, purchased from Wuhan Punosai Life Technology Co., Ltd. (Wuhan, China), were seeded into 6-well plates at a density of 1.5 × 10^5^ cells/well (6-well plate) and cultured in Proliferation Medium [DMEM (L310KJ, BasalMedia) with 10% fetal bovine serum (No.1641010-50, Pricella, Wuhan, China) and 1% penicillin/streptomycin (P/S) (No. P1400, Solarbio, Beijing, China)] in a humidified incubator with 5% CO_2_ at 37 °C. Upon reaching 70%–80% confluence (about 1 day), cells were switched to Differentiation Medium (DM: DMEM with 2% equine serum (No. ES-500, Newzerum, New York) and 1% P/S) to induce differentiation. Meanwhile, they were subjected to the following treatments: (1) Control group (cultured in DM without MRJPs); (2) MRJPs groups (cultured in DM containing MRJPs at 4, 8, or 10 mg/mL). The DM, with or without MRJPs, was refreshed every 2 days until the myoblasts reached complete differentiation.

### 2.5 Dosage regimen

According to previously reported studies, myotubes formed after 4 days of myoblast differentiation were treated with 8.0 mg/mL MRJPs, 10 μM of the muscle-atrophy inducer Dexamethasone (Dexamethasone, Dex, HY-14648, MCE) (30), and 20 μM of the mitochondrial-division/mitophagy inhibitor Mdivi-1 (Mitochondrial division inhibitor 1, Mdivi-1, HY-15886, MCE) (31, 32) for 24 h.

### 2.6 Coomassie brilliant blue staining

Initially, the cells were delicately rinsed thrice with phosphate buffer solution (PBS), followed by fixation with 4% paraformaldehyde for 15 min. Next, the paraformaldehyde was aspirated and the cells were washed twice with PBS. The PBS was completely removed and discarded. Subsequently, an appropriate amount of Coomassie Brilliant Blue Rapid Staining was added, and the cells were stained for 10 min at room temperature and protected from light, then the staining solution was removed, and the cells were washed twice with PBS. Finally, a volume of 2 mL of PBS was added to each well, and the plate was placed on an inverted fluorescence microscope (IX73 + DP80) for imaging.

### 2.7 Real-time quantitative polymerase chain reaction (RT-qPCR) analysis

Total RNA was isolated from C2C12 myotubes samples was performed using the Total RNA Extraction Kit (TIANGEN, DP430). The cDNA was generated using the HiScript III RT SuperMix for qPCR (+gDNA wiper) (R323, Vazyme). The Maxima SYBR Green qPCR Master Mix (2X) (Bimake, B21202) was used to perform quantitative real-time PCR on the LightCycler 96. Gene expression levels were quantified by the 2^−ΔΔct^ method, using *α*-actin as the internal reference.

### 2.8 Protein extraction and Western blotting

After removing the medium and rinsing twice with PBS, cells were lysed using cold RIPA buffer (Solarbio, R0010) containing freshly added phosphatase inhibitors and PMSF (Solarbio, P0100) in an ice bath for 30 min. The lysate was vortexed every 5 min. Whole cell contents were centrifuged at 4 °C for 10 min at 12,000×*g*. The supernatant was carefully transferred to a new tube. The protein concentration of each sample was determined using the BCA Protein Assay Kit. Proteins from the different samples were equally divided and loaded onto 8, 10, or 12% SDS-PAGE gels, transferred to PVDF membranes and blocked with 5% skim milk or BSA for 2 h at room temperature. The membranes were incubated overnight at 4 °C with specific primary antibodies. The membranes were washed three times with Tris-buffered saline-Tween 20 (TBST). The horseradish peroxidase-coupled secondary antibody was incubated for 1 h. Subsequently, the membrane was rinsed three times with TBST and the immunocomplexes were detected with an ECL chemiluminescent substrate kit (ABclonal, RM02867). Images were acquired using a chemiluminescent gel imaging system (Tanon, 5200). Band densities were quantified using ImageJ software.

### 2.9 ROS measurement

The treated cells were incubated with 10 μM DCFH-DA fluorescent probe at 37 °C for 20 min and gently inverted every 5 min according to the manufacturer’s protocol (Beyotime, S0033S). Afterward, the cells were rinsed thrice with serum-free media to remove extracellular DCFH-DA. Subsequently, 200 μL of a cell suspension was introduced into each well of a 96-well plate while avoiding the light. The optical density values were then measured using a fluorometric microplate reader at 488/525 nm (excitation/emission) within a 30-min timeframe.

### 2.10 JC-1 measurement

Following the manufacturer’s instructions (Beyotime, C2003S), the culture medium in the 6-well plate was removed, the cells were washed with PBS once, and 1 mL of fresh culture medium was added. Then 1 mL of JC-1 staining solution was added to each well under light-protected conditions and mixed gently. The cells were incubated at 37 °C for 20 min. After incubation at 37 °C, the supernatant was removed and washed twice with JC-1 staining buffer in the dark. Finally, 2 mL of culture medium was added. Fluorescence was then examined using a fluorescence microscope.

### 2.11 Immunofluorescence staining

The medium was discarded and the cells were washed three times with PBS. Cells were then fixed with 4% paraformaldehyde for 30 min at room temperature. After three washes with PBS for 5 min each, the cells were treated with 0.1% Triton X-100/PBS for 10 min. The cells were then washed three times with PBS 3 times for 5 min each and then blocked with 10% goat serum for 1 h at room temperature. Subsequently, cells were incubated with anti-MyHC antibody (1:200 in 10% goat serum) at 4 °C overnight. Cells were incubated with fluorescent secondary antibody (1:500) for 1 h at room temperature, protected from light. Cells were washed three times with PBS for 5 min each. Cells were stained with DAPI (Solarbio, C0065) for 10 min, then were washed three times with PBS for 5 min each. The slices were blocked with DAPI-free anti-fluorescence quencher (Solarbio, S2100). Finally, a laser confocal microscope (OLYMPUS, FV3000) was used to observe and take pictures.

### 2.12 Statistical analysis

GraphPad Prism (version 8.0.2) was used for statistical analysis. Differences between groups were analyzed using T-test and one-way ANOVA followed by Tukey’s *post-hoc* test. Experimental data were expressed as mean ± SEM. Each experiment was performed in triplicate with three wells per group. *p* < 0.05 was considered statistically significant.

## 3 Results

### 3.1 The identification of the MRJPs family

**Figure 1:**
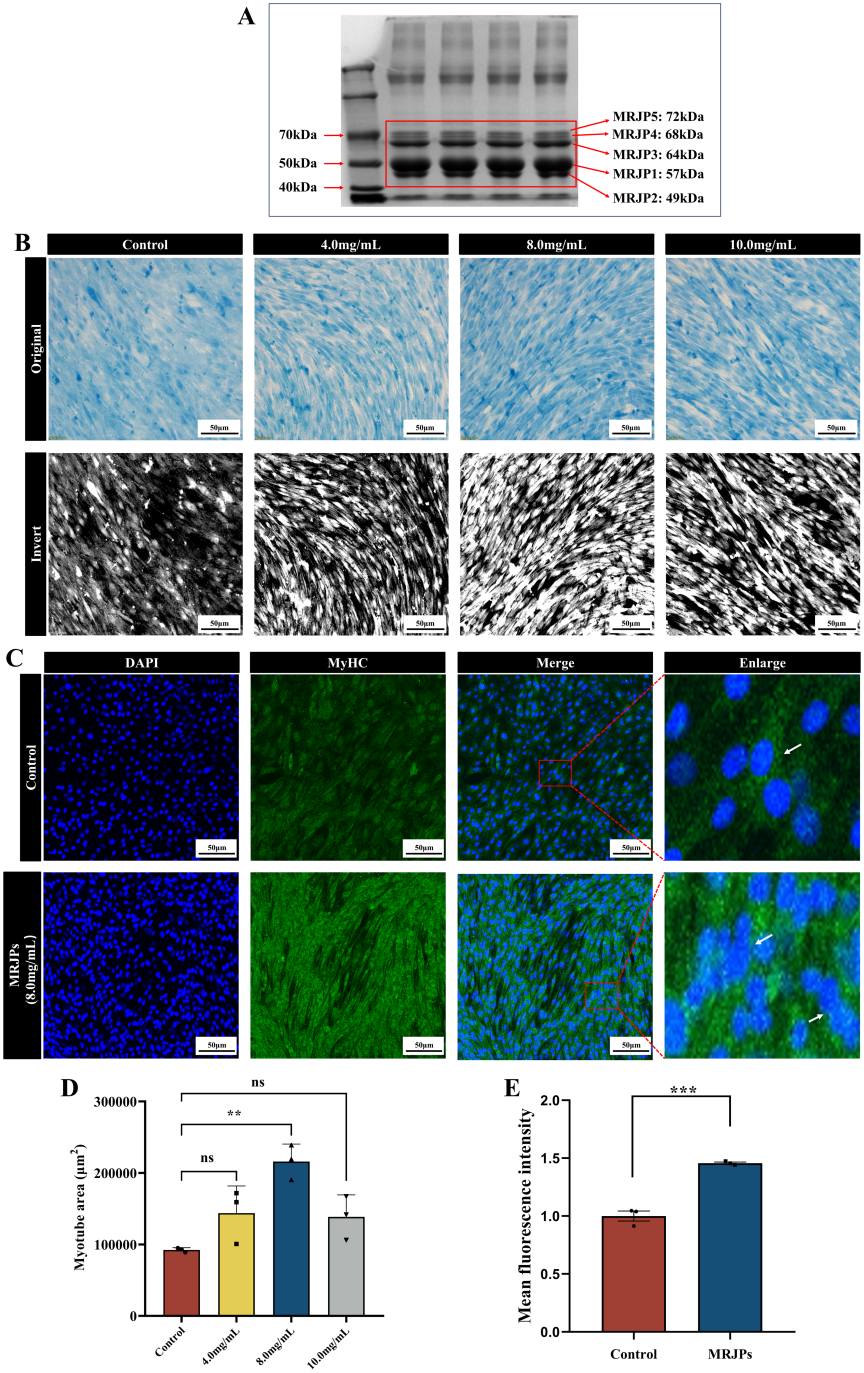
**(A)** MRJPs were separated by 8% SDS-PAGE, lane 1: Tricolor prestained protein marker (10 to 250 kDa), lane 2–5: MRJPs obtained by centrifugal dialysis. **(B,D)** The results of the Coomassie brilliant blue staining of C2C12 myoblasts differentiated with MRJPs (0, 4, 8, and 10.0 mg/mL) for 6 days and Image-J software inversion plots as well as quantitative analysis of myotubes (images were taken using an Olympus IX73 + DP80 inverted fluorescence microscope system with a magnification of 200×; scale bar = 50 μm). **(C,E)** Immunofluorescence staining of MyHC (green) positive C2C12 myoblasts differentiated with 8.0 mg/mL MRJPs for 6 days (images were taken using an Olympus FV3000 confocal laser scanning microscope system, magnification 200×; scale bar = 50 μm). MRJPs, Major Royal Jelly Proteins. The white arrow points to the multi-nuclear myotube formed by cell fusion. **P < 0.01, ***P < 0.001.

According to the result of SDS-PAGE (Figure 1A), there are five conspicuous bands in the lane. Based on the previous analysis and the molecular weight differences of MRJPs, it is speculated that the five bands, from top to bottom, correspond to MRJP5 (72 kDa), MRJP4 (68 kDa), MRJP3 (64 kDa), MRJP1 (57 kDa), and MRJP2 (49 kDa).

### 3.2 Effect of MRJPs on C2C12 myotubes in morphology

Based on other research (33) and our previous studies (34), it was concluded that a 6-day period of myotube differentiation is the most effective. To identify the most effective treatment concentration of MRJPs, this study administered MRJPs at concentrations ranging from 4 to 10 mg/mL to C2C12 myoblasts for a duration of 6 days. In this study, the effects of MRJPs on the differentiation of C2C12 myoblasts into myotubes were investigated at the morphological level by using Coomassie brilliant blue staining and MyHC immunofluorescence. The objective was to determine whether the myotube differentiation model was successfully established. Subsequently, we utilized Image-J software to invert the images to more clearly visualize the changes (Figure 1B). The findings indicated a marked increase in myotube formation in the MRJPs group compared to the control group, particularly in the 8.0 mg/mL MRJPs group (Figures 1B,D). In addition, 8.0 mg/mL MRJPs significantly increased the density and length of C2C12 myotubes (Figures 1C,E). These results indicate that MRJPs can promote the differentiation of C2C12 myoblasts into myotubes.

### 3.3 Effects of MRJPs on C2C12 myotubes in myogenic factors

**Figure 2:**
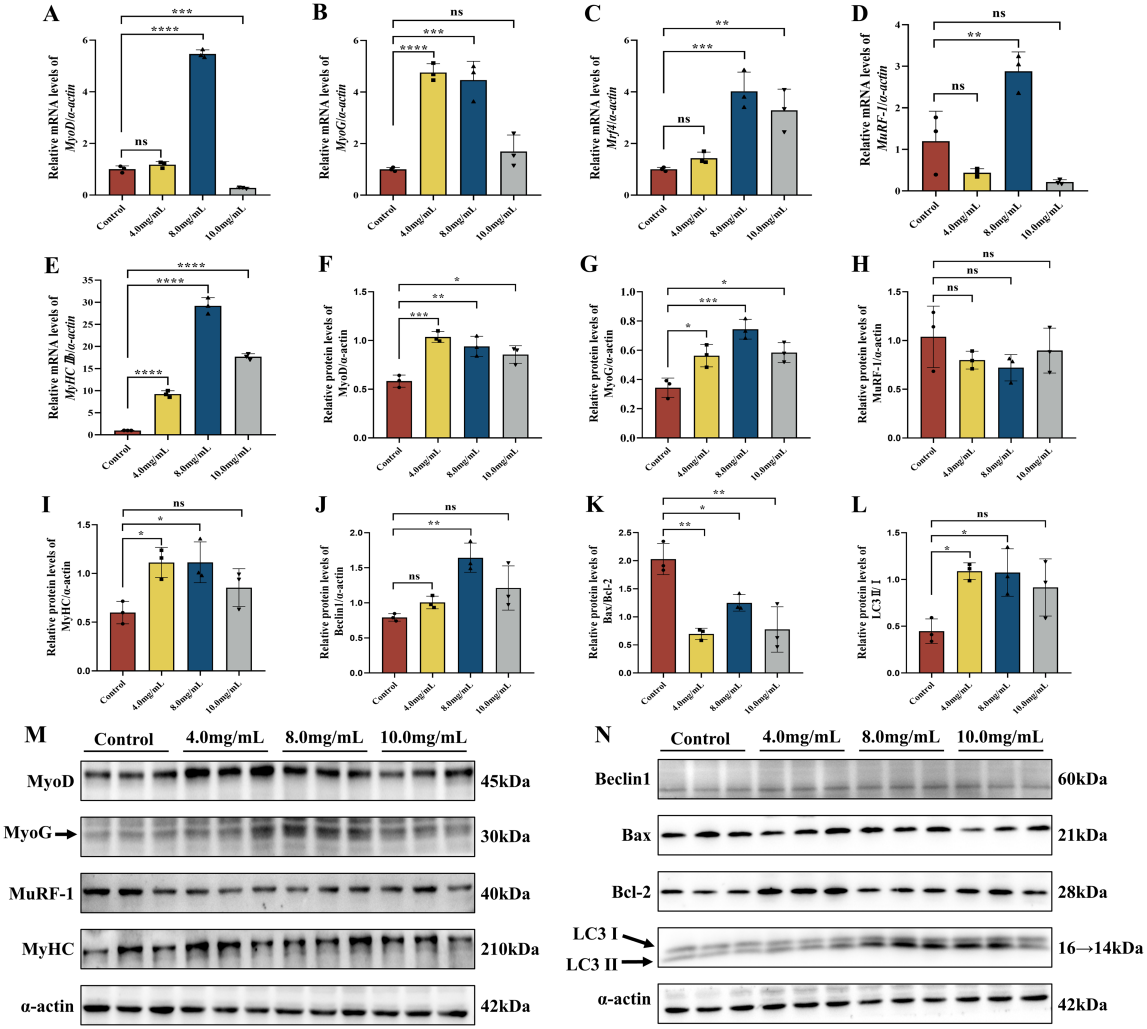
**(A–E)** mRNA expression levels of *MyoD*, *MyoG*, *Mrf4*, *MuRF-1*, and *MyHC IIb* in C2C12 myoblasts differentiated with MRJPs (0, 4, 8, 10.0 mg/mL). *n* = 3 per group. **(F–I,M)** Protein expression levels of MyoD, MyoG, MuRF-1, and MyHC in C2C12 myoblasts differentiated with MRJPs (0, 4, 8, and 10.0 mg/mL). *n* = 3 per group. **(J–L,N)** Protein expression levels of LC3, Bax, Bcl-2, and Beclin1 in C2C12 myoblasts differentiated with MRJPs (0, 4, 8, and 10.0 mg/mL). *n* = 3 per group. All expressions were normalized to α-actin. MRJPs, Major Royal Jelly Proteins; MyoD, Myogenic Differentiation; MyoG, Myogenin; MuRF-1, Muscle Ring Finger 1; Bcl-2, B-cell lymphoma-2; Bax, Bcl-2-Associated X; LC3, Microtubule-associated protein 1 light chain 3. *P < 0.05, **P < 0.01, ***P < 0.001, ****P < 0.0001.

To evaluate the effect of MRJPs on myogenic factors during C2C12 myotube differentiation, key muscle growth markers (*MyoD*, *MyoG*, *Mrf4* and *MyHC IIb*) and the muscle atrophy marker muscle ring finger 1 (*MuRF-1*) were examined in C2C12 myotubes exposed to MRJPs for 6 days. RT-qPCR results showed that compared with the control group, the mRNA expression levels of *MyoD*, *MyoG*, *Mrf4* and *MyHC IIb* in 8.0 mg/mL MRJPs group were significantly upregulated (Figures 2A–C,E), and the protein expression levels of MyoD, MyoG and MyHC were consistent with the corresponding mRNA levels (Figures 2F,G,I,M). It is worth noting that although MuRF-1 mRNA was elevated, MuRF-1 protein remained unchanged compared with the control group (Figures 2D,H,M). This study showed that MRJPs induced expression of muscle-growth markers during differentiation. However, it had no significant effect on the muscle atrophy protein MuRF-1. This may be because the size of muscle fibers is influenced by their ability to proliferate and is controlled by a coordinated balance of protein synthesis and breakdown.

### 3.4 Effects of MRJPs on C2C12 myotubes in apoptosis and autophagy

LC3I is a cytoplasmic form that is converted to the autophagosome-associated LC3-II. Therefore, the LC3II/I ratio was used to measure autophagy activity. Western blotting and quantitative analysis showed that the LC3-II/I ratio and the autophagy associated protein Beclin1 increased after 6 days of MRJPs treatment in myoblasts compared to the control group (Figures 2J,L,N). In particular, the increase was more pronounced in the 8.0 mg/mL MRJPs group. Following MRJP treatment, the Bax/Bcl-2 ratio decreased (Figures 2K,N). The results indicated that MRJPs increased autophagy and reduced apoptosis of cells, stimulating the autophagic degradation process of cells. In short, MRJPs enhance differentiation by activating autophagy, inhibit apoptosis, and maintain C2C12 myoblast homeostasis.

### 3.5 Effect of MRJPs on C2C12 myotubes in mitochondrial biogenesis and uncoupling

**Figure 3:**
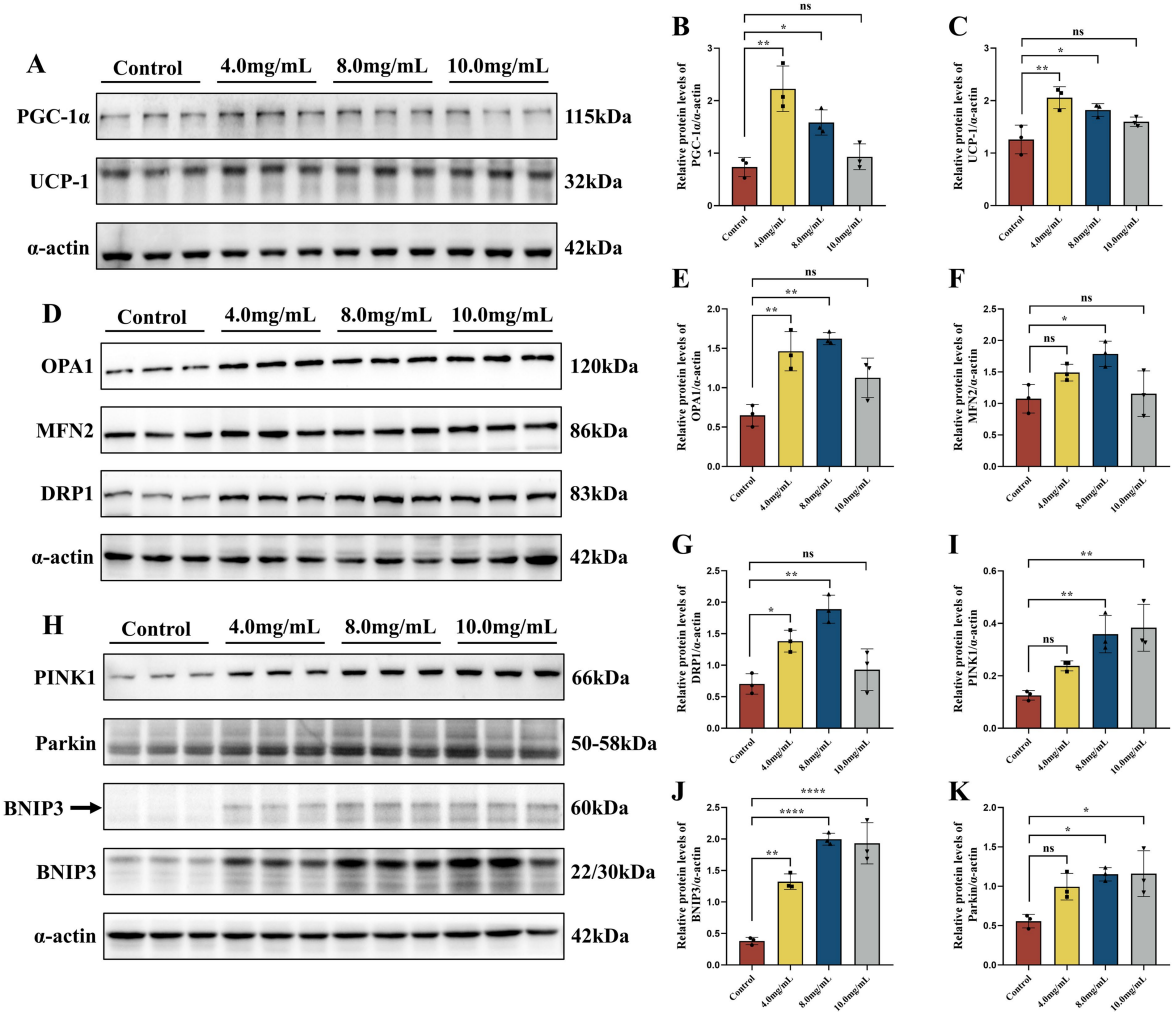
**(A–C)** Protein expression levels of PGC-1α and UCP-1 in C2C12 myoblasts differentiated with MRJPs (0, 4, 8, and 10.0 mg/mL). *n* = 3 per group. All expressions were normalized to α-actin. **(D–K)** Protein expression levels of OPA1, MFN2, DRP1, PINK1, Parkin and BNIP3 in C2C12 myoblasts differentiated with MRJPs (0, 4, 8, and 10.0 mg/mL). *n* = 3 per group. All expressions were normalized to α-actin. MRJPs, Major Royal Jelly Proteins; PGC-1α, Peroxisome Proliferator-activated Receptor Gamma Coactivator-1 Alpha; UCP-1, Uncoupling Protein 1; OPA1, Optic Atrophy 1; MFN2, Mitofusin 2; DRP1, Dynamin-related protein 1; PINK1, PTEN induced putative kinase 1; Parkin, PARK2, RBR E3 ubiquitin protein ligase Gene; BNIP3, Bcl-2 interacting protein 3. *P < 0.05, **P < 0.01, ****P < 0.0001.

Mitochondrial function is an important determinant of skeletal muscle function. MRJPs increased mitochondrial content during the differentiation of C2C12 myoblasts. PGC-1α is closely related to mitochondrial biogenesis. The UCP-1 protein attenuates mitochondrial ROS production by inducing mild uncoupling, a process linked to longevity. Western blotting results showed that PGC-1α and UCP-1 protein expression in MRJPs differentiated for 6 days were significantly higher than in the control group (Figures 3A–C). Thus, MRJPs augment the expression of key mitochondrial biogenesis and uncoupling proteins during myoblast differentiation.

### 3.6 Effects of MRJPs on C2C12 myotubes in mitochondrial dynamics and mitophagy

To further explore the potential mechanism by which MRJPs promotes differentiation of C2C12 myoblasts, mitochondrial dynamics and mitophagy were investigated. In this study, the protein expression levels of OPA1, MFN2, DRP1, PINK1, Parkin, and BNIP3 were significantly increased in C2C12 myoblasts treated with MRJPs for 6 days compared with the control group (Figures 3D–K), especially in the 8.0 mg/mL MRJPs group. Interestingly, after 6 days of MRJPs treatment in C2C12 myoblasts, the protein expression levels of OPA1, MFN2, DRP1, PINK1, Parkin, and BNIP3 in mitochondria isolated from myotubes were not significantly different from the control group, but showed a tendency to increase (Supplementary material).

### 3.7 Effects of MRJPs on C2C12 myotubes in mitochondrial function

**Figure 4:**
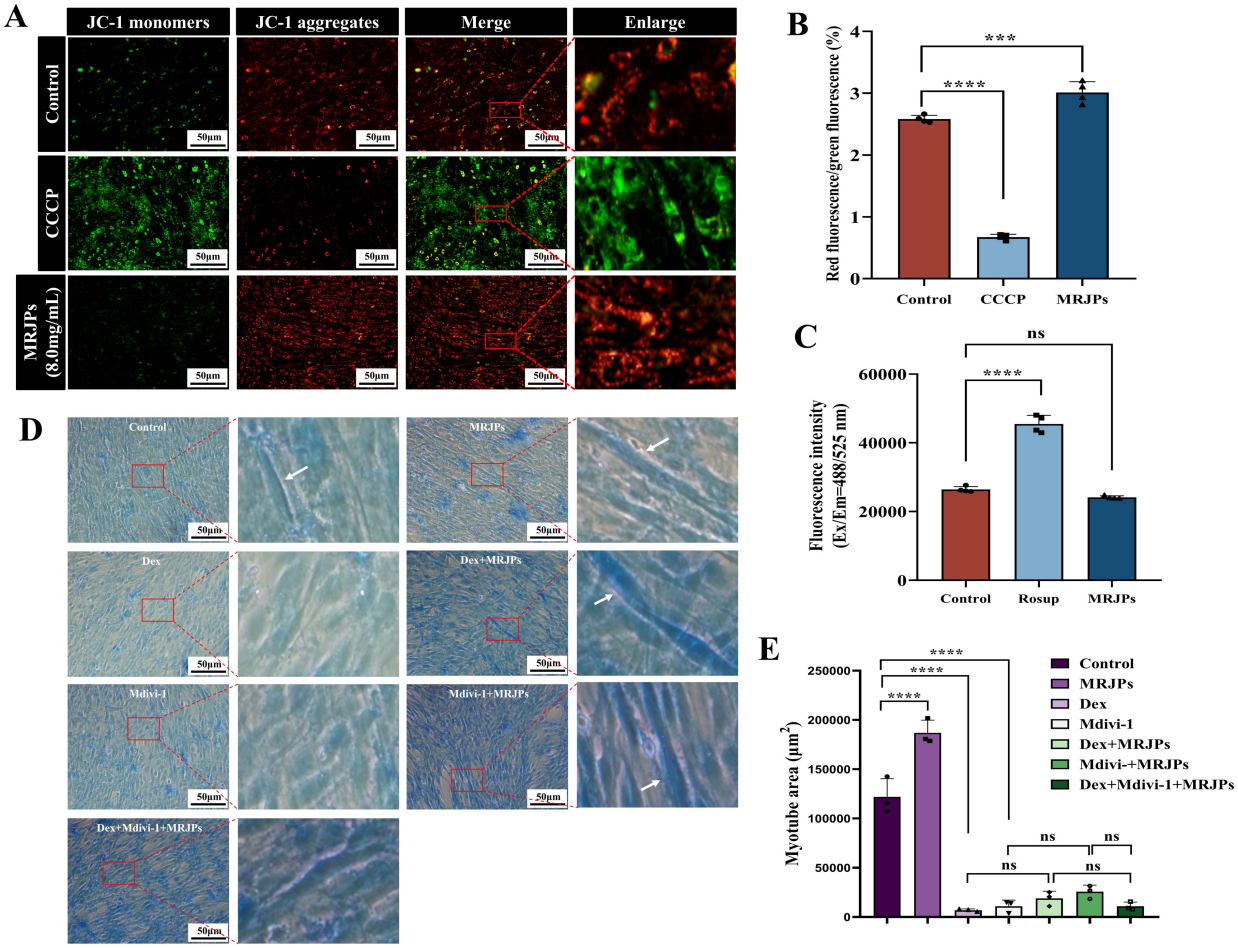
**(A–C)** The fluorescence intensity of JC-1 was taken by Olympus IX73 + DP80 inverted fluorescence microscope system and the absorbance of ROS was analysed by Multifunctional Fluorescent Enzyme Labeler (Tecan, Spark) in C2C12 myoblasts differentiated with 8.0 mg/mL MRJPs for 6 days, with a magnification of 200×; scale bar = 50 μm. *n* = 3 per group. **(D,E)** The results of the Coomassie brilliant blue staining of myotubes after 4 days of C2C12 myoblasts differentiation were treated with 8.0 mg/mL MRJPs and 10 μM Dex and 20 μM Mdivi-1 for 24 h. *n* = 3 per group. MRJPs, Major Royal Jelly Proteins; Dex, Dexamethasone; Mdivi-1, Mitochondrial division inhibitor 1; CCCP, Positive control; Red represents JC-1 polymer; Green represents JC-1 monomer. The white arrows indicate the representative muscular tubes formed by cell fusion. ***P < 0.001, ****P < 0.0001.

To determine whether oxidative stress induces mitochondrial dysfunction, fluorescence intensity of ROS, JC-1 aggregates and monomers was measured by fluorescence microscopy. This study found that after 6 days of MRJPs treatment in C2C12 myoblasts, JC-1 staining revealed increased red fluorescence and decreased green fluorescence in the 8.0 mg/mL MRJPs group compared with the control group and the positive control group (Figures 4A,B). The JC-1 aggregate/monomer ratio increased significantly. ROS levels in both the control and 8.0 mg/mL MRJPs groups were markedly lower than in the positive control group. Interestingly, ROS did not change significantly in the 8.0 mg/mL MRJPs group compared to the control group (Figure 4C). Collectively, these results indicate that MRJPs improve mitochondrial function and thereby promote C2C12 myotube formation.

### 3.8 Dex successfully induced muscle atrophy and Mdivi-1 successfully inhibited mitochondrial division and mitophagy

To further verify whether MRJPs promote myotube differentiation through mitochondrial division and mitophagy, and whether MRJPs can reverse Dexamethasone-induced myotube atrophy by reversing mitochondrial division and mitophagy, 8.0 mg/mL MRJPs, 10 μM Dex or 20 μM Mdivi-1 were simultaneously applied to 4-day differentiated myotubes for 24 h. The Coomassie brilliant blue staining showed that the myotube area of Dex group and Mdivi-1 group was significantly reduced compared with the control group (Figures 4D,E).

**Figure 5:**
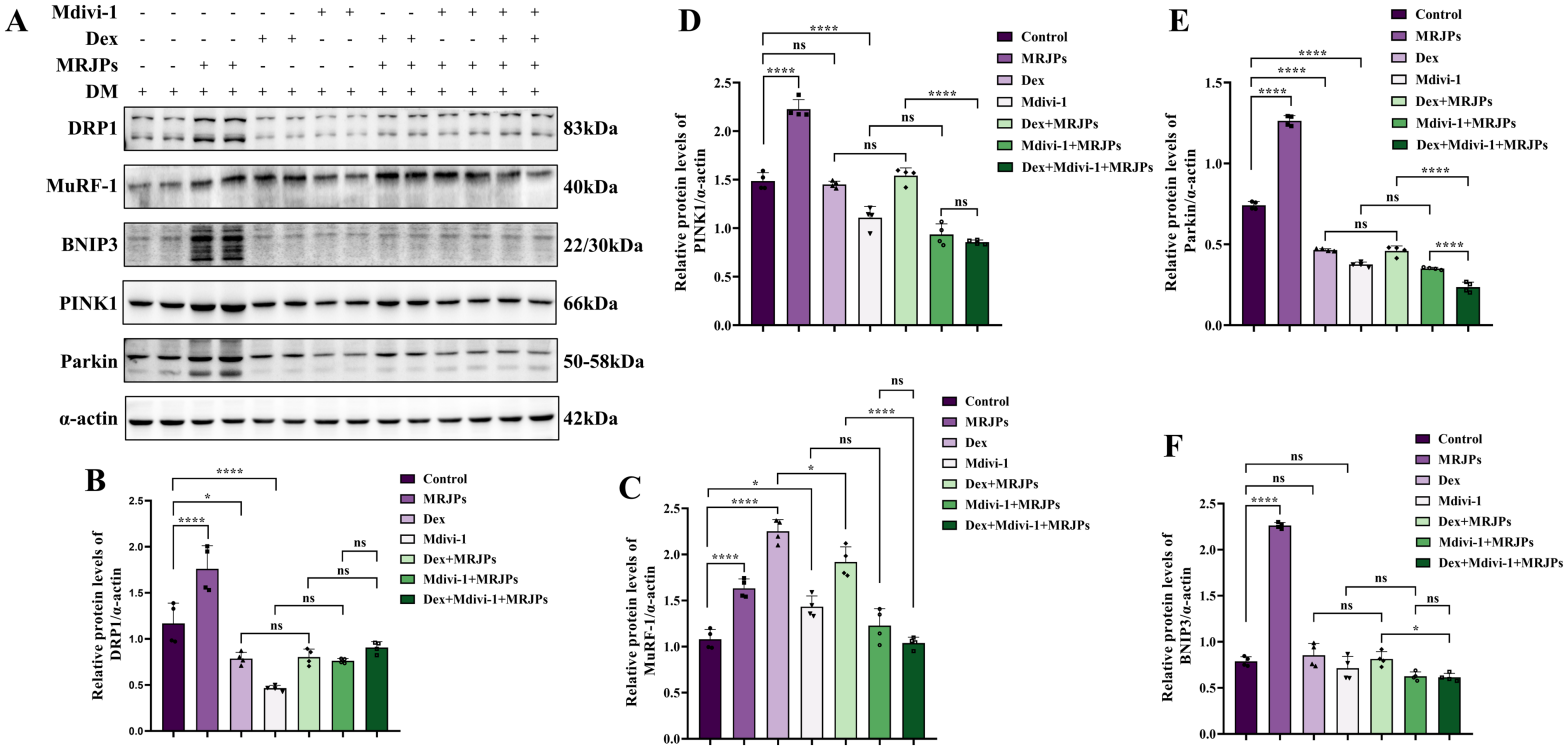
**(A–F)** Protein expression levels of DRP1, MuRF-1, BNIP3, PINK1, and Parkin in myotubes after 4 days of C2C12 myoblasts differentiation were treated with 8.0 mg/mL MRJPs and 10 μM Dex or 20 μM Mdivi-1 for 24 h. *n* = 3 per group. All expressions were normalized to α-actin. DM, Differentiation Medium; MRJPs, Major Royal Jelly Proteins; Dex, Dexamethasone; Mdivi-1, Mitochondrial division inhibitor 1. *P < 0.05, ****P < 0.0001.

Moreover, MuRF-1 protein expression level was significantly increased, while DRP1, PINK1, Parkin, and BNIP3 protein expression levels were significantly decreased (Figures 5A–F). These results suggest that Dex and Mdivi-1 successfully induced changes in myotubes. Interestingly, when Dex or Mdivi-1 was co-administered with 8.0 mg/mL MRJPs, although there was no significant change in myotube area and protein expression levels of DRP1, PINK1, Parkin, and BNIP3, they all tended to increase. The expression level of MuRF-1 protein decreased. These trends are consistent with the morphology that myotubes showed a tendency to recover after co-treatment with Dex or Mdivi-1 and 8.0 mg/mL MRJPs (Figure 4D). These results indicated that MRJPs promoted C2C12 myotube differentiation through mitochondrial division and mitophagy, but did not reverse myotube atrophy.

### 3.9 Effect of MRJPs on muscle growth markers and apoptosis of C2C12 myotube treated with Dex and Mdivi-1

**Figure 6:**
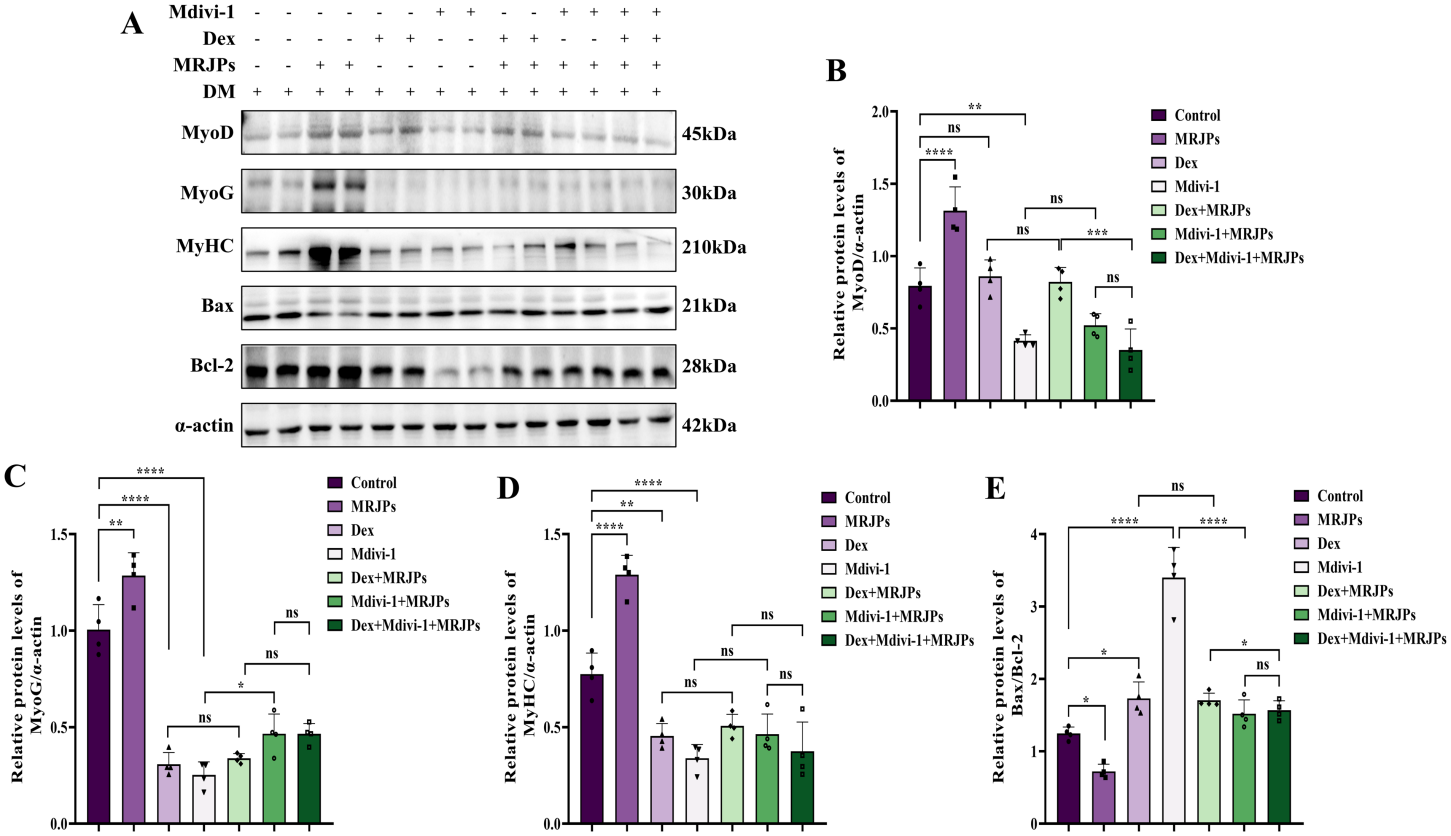
**(A–E)** Protein expression levels of MyoD, MyoG, MyHC, and Bax/Bcl-2 in myotubes after 4 days of C2C12 myoblasts differentiation were treated with 8.0 mg/mL MRJPs and 10 μM Dex or 20 μM Mdivi-1 for 24 h. *n* = 3 per group. All expressions were normalized to α-actin. DM, Differentiation Medium; MRJPs, Major Royal Jelly Proteins; Dex, Dexamethasone; Mdivi-1, Mitochondrial division inhibitor 1. *P < 0.05, **P < 0.01, ***P < 0.001, ****P < 0.0001.

After simultaneous treatment with 8.0 mg/mL MRJPs plus 10 μM Dex or 20 μM Mdivi-1 for 24 h, Western blotting results showed that the protein expression of MyoD, MyoG, and MyHC in the Dex group and the Mdivi-1 group were significantly decreased compared with the control group (Figures 6A–D). The ratio of Bax/Bcl-2 also increased significantly (Figures 6A,E). This further indicated that MRJPs promoted the increase of C2C12 muscle growth factors and decreased cell apoptosis through mitochondrial division and mitophagy, thus promoting C2C12 myotube differentiation. The protein expression levels of MyoD, MyoG, MyHC, and Bax/Bcl-2 did not change significantly when cells were co-treated with Dex and 8.0 mg/mL MRJPs. It is worth noting that after co-treatment with Mdivi-1 and 8.0 mg/mL MRJPs, MyoG protein expression increased and Bax/Bcl-2 ratio decreased. This is consistent with previous results demonstrating that MRJPs can counteract the inhibition of mitochondrial division and mitophagy to restore muscle growth and inhibit apoptosis.

### 3.10 Effects of MRJPs on autophagy and mitochondria-related factors of C2C12 myotubes treated with Dex and Mdivi-1

**Figure 7:**
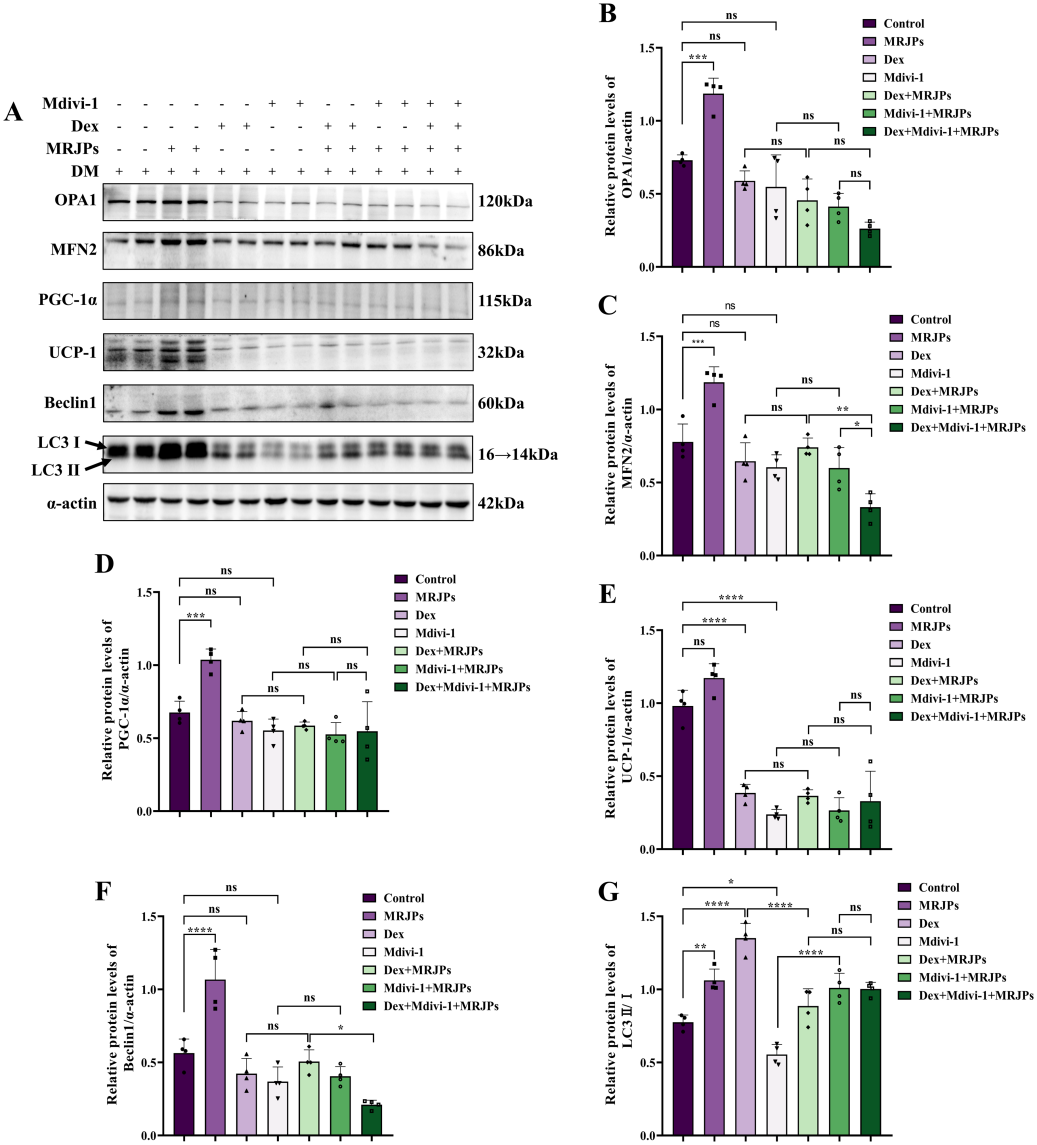
**(A–G)** Protein expression levels of OPA1, MFN2, PGC-1α, UCP-1, Beclin1, and LC3II/I in myotubes after 4 days of C2C12 myoblasts differentiation were treated with 8.0 mg/mL MRJPs and 10 μM Dex or 20 μM Mdivi-1 for 24 h. *n* = 3 per group. All expressions were normalized to α-actin. DM, Differentiation Medium; MRJPs, Major Royal Jelly Proteins; Dex, Dexamethasone; Mdivi-1, Mitochondrial division inhibitor 1. *P < 0.05, **P < 0.01, ***P < 0.001, ****P < 0.0001.

After simultaneous treatment with 8.0 mg/mL MRJPs plus 10 μM Dex or 20 μM Mdivi-1 for 24 h, Western blotting results showed that the protein expression levels of OPA1, MFN2, PGC-1α and Beclin1 in the Dex group and the Mdivi-1 group did not differ significantly compared with the control group (Figures 7A–D,F). In the Dex group, UCP-1 expression decreased significantly and LC3II/I ratio increased significantly, likely resulting from Dex-induced apoptosis (Figures 7A,E,G). The UCP-1 protein expression and LC3II/I ratio in Mdivi-1 group decreased significantly, which may be related to the inhibition of mitophagy by Mdivi-1, leading to disruption of mitochondrial homeostasis (Figures 7A,G). Compared with Dex group or Mdivi-1 group, there were no significant changes in the protein expression levels of OPA1, MFN2, PGC-1α, UCP-1, and Beclin1 when cells were co-treated with Dex or Mdivi-1 plus 8.0 mg/mL MRJPs (Figures 7A–F). Interestingly, LC3II/I ratio increased after the addition of 8.0 mg/mL MRJPs (Figures 7A,G). This suggests that MRJPs have the potential to improve the imbalance of autophagy and thus inhibit apoptosis.

## 4 Discussion

Skeletal-muscle development involves clonal proliferation of myoblasts, their directed differentiation, and fusion into multinucleated myotubes, which eventually develop into mature myofibers (35). Myofibers are the basic contractile units of skeletal muscle and can adopt distinct phenotypes (36). Four MyHC isoforms have been identified (37). In adult rodent skeletal muscle, MyHC IIb is the fastest isoform, conferring the highest shortening velocity and lowest oxidative capacity (36). Species differences constrain the extrapolation of C2C12 data to the complexity of human muscle. For example, primary human myoblasts (PHMs) and rat myoblasts (L6) exhibit divergent cell-cycle kinetics and differentiation profiles relative to C2C12 cells (38, 39). Although C2C12 cells offer valuable insights, confirmation in PHMs and other human-relevant *in vitro* systems is essential for translational validity.

Myogenic differentiation is a controlled process that is governed by Myogenic regulatory factors (MRFs), such as MyoD, MyoG, Myf5, and Mrf4 (40). The *MyoD* and *Myf5* genes primarily contribute to the differentiation of precursor myoblasts that form muscle fibers and the proliferation of myoblasts. On the other hand, the *MyoG* and *Myf6* genes are involved in the fusion and differentiation of myoblasts (41). Mrf4 is activated in the later stages of embryonic muscle development and is quickly increased during the differentiation of myoblasts in a laboratory setting, when they fuse to form create multinucleated myotubes (42). C2C12 myoblast differentiation involves sequential marker dynamics, in which transcription factors (MyoD), structural proteins (MyHC), and mitochondrial metabolic factors (PGC-1α) are core regulatory nodes.

Unlike other research that has found that resveratrol (43), fucoxanthin (44), delphinidin (45), myricanol (30), and trimetazidine (46) inhibit Dexamethasone-induced myotube MuRF-1 expression. Alternatively, our results are consistent with previous studies that RJ does not ameliorate MuRF-1 expression associated with muscle atrophy, but it does increase the expression of genes associated with muscle production (47). This is also related to the fact that MRJPs did not rescue Dex-induced muscle atrophy in the follow-up study. Although the present study and several previous reports suggest that MRJPs have a more favorable effect on the proliferation and differentiation of myoblasts (48), the results reported herein do not deny the possibility that some of the effects of MRJPs are related to MuRF-1. These results suggest that MRJPs can promote myotube differentiation by improving mitochondrial function, but does not mitigate Dex-induced muscle atrophy. Indeed, it is possible that the prolonged action of Dexamethasone and mitophagy inhibitors is responsible for excessive cell death. This suggests that MRJPs can be used as potential modulators of myotube formation, but may not be effective therapeutic agents for sarcopenia. But our 24-h treatment window constitutes an acute model and may not fully recapitulate the chronic muscle atrophy seen clinically.

Existing studies are mostly based on cellular and animal models, and clinical studies are urgently needed to validate their efficacy in human sarcopenia. The synergistic effects of MRJPs with other anti-sarcopenia targets warrant further exploration in the future, and the delivery system needs to be optimized to improve targeting.

Mitochondria serve as the primary energy generators of the cell and play a crucial role in controlling many cellular processes. Furthermore, due to the abundance of mitochondria in skeletal muscle, the quantity and performance of mitochondria play a crucial role in preserving muscle mass and functionality. Notably, C2C12 myoblast differentiation may be associated with oxidative stress (49). Nevertheless, there have been limited investigations on the impact of MRJPs on oxidative stress in myoblasts. Although our results showed minimal changes in ROS, levels tended to decrease, whereas MMP tended to increase. But DCFH-DA lacks ROS specificity; it can be oxidized by RNS, peroxidases, cytochrome c, and other reactive species. This limitation is exacerbated in skeletal muscle, where abundant mitochondria and oxidant-generating enzymes amplify background oxidation. Additionally, DCFH-DA autoxidizes spontaneously when exposed to light in culture, and its fluorescent product (DCF) can photosensitize further ROS generation (50). Single-endpoint readings, as used here, cannot resolve transient bursts from sustained low-level ROS generation. The 30-min recording window risks probe depletion or saturation, and DCF fluorescence is non-linear with ROS concentration because of pH, redox status, and metal-ion interference.

The balance between autophagy and apoptosis is also critical for maintaining skeletal muscle mass (51). Loss of autophagy or apoptosis leads to organelle dysfunction, protein misfolding, and ROS accumulation, resulting in skeletal muscle atrophy in various diseases (52). It is well known that LC3 is essential for early autophagic vesicle membrane formation (51). Our study demonstrated that MRJPs intervention significantly increased the expression of LC3II/I and Beclin1 proteins, activating the autophagy signaling pathway to clear misfolded proteins and damaged organelles. Our results, together with several other studies, indicate that removal of dysfunctional or damaged mitochondria is essential for improving mitochondrial function. Decrease in MMP is a hallmark event in early apoptosis (53). In our study, the rise in MMP enabled MRJPs to significantly increase the JC-1 aggregate/monomer ratio and decrease Bax/Bcl-2 expression. JC-1 responses are concentration-dependent and cell-type-specific. The red/green fluorescence ratio is non-linear and cannot be predicted *a priori*. Single-endpoint readings miss ΔΨm dynamics and lack correction for uneven probe loading or inter-cell variability. Although spontaneous contractions could artifactually alter ΔΨm, none were detected under our experimental conditions.

The brown-adipose mitochondrial uncoupling protein 1 (UCP-1) regulates energy metabolism and mitochondrial homeostasis by uncoupling oxidation and phosphorylation, which deplete the electron transport chain’s proton gradient to create heat (54). UCP-1 modulates ROS generation; however, ROS and ROS-derived reactive aldehydes can reciprocally activate uncoupling proteins, leading to a decline in MMP (55). PGC-1α is a coactivator of peroxisome proliferator-activated receptor-*γ* (PPAR-γ), which plays an important role in regulating *de novo* mitochondrial biogenesis. We found that MRJPs improved mitochondrial biogenetic PGC-1α protein expression during myotube differentiation.

PGC-1α controls mitochondrial function by changing genes regulating mitochondrial biogenesis and dynamics (56). Mitochondrial fusion is facilitated by two proteins, Mitochondrial proteins 1 and 2 (MFN1 and MFN2), which are found in the outer mitochondrial membrane, and optic atrophy 1 (OPA1), which is positioned in the inner mitochondrial membrane (57). Mitochondrial fission is regulated by the proteins DRP1. Mitochondria are organelles that undergo continuous structural remodeling through fusion and fission events. The expression of MFN2 is highly prevalent in skeletal muscle. Muscle MFN2 loss causes mtDNA depletion, OXPHOS impairment, mitochondrial membrane potential reduction, and oxidative stress (58). Our results demonstrated that MRJPs facilitated mitochondrial fusion and fission by upregulating MFN2, OPA1, and DRP1 expression. Furthermore, it has been demonstrated that PGC-1α can stimulate the expression of MFN2 in myoblasts (59). According to our results, MRJPs enhance mitochondrial homeostasis to maintain mitochondrial homeostasis. Moreover, there could be a correlation between mitochondrial biogenesis and the dynamics of existing mitochondria (60). These findings suggest that MRJPs may affect myotube differentiation by regulating mitochondrial production and movement and increasing PGC-1α protein expression.

Mitophagy maintains the health of the mitochondrial network by removing damaged mitochondria. In skeletal muscle, decreased mitophagy may lead to the accumulation of damaged mitochondria, which in turn triggers muscle dysfunction and atrophy. If the PINK1-Parkin pathway malfunctions, it can cause mitochondrial fragmentation occurs aberrantly and damaged mitochondria to stay in cells instead of being removed. This builds up defective mitochondria in myoblasts, thereby exacerbating sarcopenia. BNIP3 and NIX are autophagy receptors located on the outer membrane of mitochondria. They cause mitochondrial depolarization, attach to LC3, and link mitochondria to autophagosomes (61). In contrast, BNIP3 and NIX bind to Bcl-2 and disrupt the interactions between Beclin1 and Bcl-2, thus enabling Beclin1 to initiate mitophagy. In summary, MRJPs promoted myotube differentiation by increasing the expression of mitochondria-associated proteins after treating C2C12 myoblasts for 6 days. The centrality of mitochondria in skeletal muscle is reflected in the multidimensional aspects of energy supply, quality control, and disease regulation. Abnormal mitochondrial function not only directly leads to sarcopenia, but is also closely related to metabolic diseases (e.g., obesity, diabetes). Future research should investigate the metabolic crosstalk between mitochondria and other organs, and devise integrated, multimodal interventions—such as combined exercise, pharmacological, and nutritional strategies—to enhance skeletal muscle health and clinical outcomes.

Picard et al. showed that mitochondrial isolation can significantly alter mitochondrial function, including heightened Ca^2+^-induced mPTP sensitivity, perturbed respiratory coupling, and elevated ROS emission (62). Thus, isolated mitochondria may inadequately recapitulate the bioenergetic status of mitochondria *in situ*. In our research, several trend-level effects may reach significance with greater statistical power. Resource and experimental constraints limited us to a sample size that was feasible yet underpowered for small effects.

Glucose concentration critically affects muscle differentiation and mitochondrial function. High glucose suppresses myogenesis by down-regulating MyoD, myogenin, and AKT phosphorylation, whereas low glucose similarly blunts these markers and lowers PGC-1α (63). Because MyoD, MyoG, MyHC, PGC-1α, and LC3-II/I are all glucose-sensitive, our findings may be modulated by extracellular glucose. Although high-glucose DMEM was refreshed every 48 h, continuous glucose monitoring or defined supplementation would refine interpretation. Future work should adopt simultaneous real-time recordings of ΔΨm and glucose flux to yield more reliable, dynamic data.

## 5 Conclusion

In this study, MRJPs were found to promote the differentiation of C2C12 myoblasts to form myotubes, but failed to reverse Dex-induced acute muscle atrophy. The underlying mechanism appears to involve MRJP-mediated reductions in apoptosis, enhanced autophagy, stimulated mitochondrial biogenesis, and preservation of both mitochondrial homeostasis and mitophagy, and avert the loss of mitochondrial membrane potential. MRJPs may be used as an active ingredient to promote differentiation function of skeletal muscle by enhancing mitochondrial function.

## 6 Limitation

Because myotube differentiation of C2C12 cells is a complex process governed by multiple interrelated stressors, more research is needed to elucidate additional effects of MRJPs on myotube differentiation and the underlying mechanisms. This study only confirmed *in vitro* experiments that MRJPs promote the differentiation and myotube formation in C2C12 myoblasts and its possible mechanism, which must be validated in future *in-vivo* studies, in order to provide further evidence for the development of functional foods with MRJPs as the main component. Clinical muscle atrophy is usually chronic, but our model is acute, which may still be a long way from achieving clinical value.

## Data Availability

The original contributions presented in the study are included in the article/Supplementary material, further inquiries can be directed to the corresponding authors.
