# Supplementary material for: Major Royal Jelly Proteins promote C2C12 myotubes differentiation by improving mitochondrial function
Source: Front Nutr. 2026 Jan 5;12:1636751. doi: 10.3389/fnut.2025.1636751 (PMC12812747; doi:10.3389/fnut.2025.1636751)
Supplement: Supplementary file 1 [file Data_Sheet_1.pdf]

1. Materials and Methods

1.1 Extraction of mitochondria from myotubes

Treated cells were incubated with Mitochondrial Separation Reagent (C3601) on ice for 15 min, vortexed at 5-min intervals, and manipulated according to the protocol of the manufacturer (Beyotime, Shanghai, China). Then, mitochondrial proteins were lysed with lysate and protein concentration was determined using the Bradford method (P0006C).

1.2 Real Time - quantitative Polymerase Chain

The cells were cultured in a six-well plate. Total RNA was extracted using the TRIzol method (Invitrogen). Each RNA sample was reverse transcribed with All-in-One cDNA Synthesis SuperMix (TaKaRa) according to the manufacturer's protocol. The qRT-PCR was performed with qPCR SYBR Green PCR Master Mix (TaKaRa) and specific mouse primers. The first step is to remove genomic DNA: Random Primer (6 mer) 1μL, dNTP Mix (10nm) 1μL, template RNA 2000 ng, RNase-free ddH$_2$O supplemented 10 μL, 65℃ for 5 min, 4℃ for 2 min; Step 2 Reverse transcriptional reaction: 4 μL 5X Primer Script Buffer, 0.5 μL RNase Inhibitor, 1 μL Primer Script Reverse Transcriptase and 4.5 μL RNase-free ddH2O were added into the reaction products of the first step. The cDNA was synthesized using the following reaction conditions: 30℃ for 10 min; 42 ℃ 60 min; 70 ℃ for 15 min. The product was stored at -80℃.
QPCR was performed using 2X SYBR Green qPCR Mix (SparkJade, Bio, China) on a Lightcycler 96 to confirm the relative levels of expression of genes in the C2C12 myotubes. The total volume of the PCR reaction was 10 μL, containing 0.5 μL of each primer (10μM), 1 μL cDNA, 5 μL 2X SYBR Green qPCR Mix, 3 μL RNase-free ddH2O. PCR cycling conditions were as follows: initial 5 min denaturation at 95℃, followed by 45 cycles of amplification at 95℃ for 10 sec, 60℃ for 10 sec and 72℃ for 15 sec. The data were analyzed by the $2^{-\Delta\Delta Ct}$ threshold cycle method and normalized to α-actin. Sequences of the forward and reverse primers used are shown in **Table 1**.

Table 1: RT-qPCR primer sequences

Primer Name	Primer sequence (5′-3′)
α-actin	F：TATCGGTATGGAGTCTGCGG
	R：CACACTGAGTACTTGCGCTC
MyHCIIb	F：CTCTTCCCGCTTTGGTAAGTT
	R：CAGGAGCATTTCGATTAGATCCG
MyoD	F：ATGATGACCCGTGTTTCGACT
	R：CACCGCAGTAGGGAAGTGT
MyoG	F：GCAGGCTCAAGAAAGTGAAT

	R：TAGGCGCTCAATGTACTGGAT
Mrf4	F：CCTCAGCCTCCAGCAGTCTT
	R：TACTTCTCCACCACCTCCTCCA
MuRF-1	F：CATTGTGTGACTGGCGATTGT
	R：TCTCTAGGCCACCGAGTGAGA

1.3 Western Blotting

Table 2: Antibody name and part number

Antibody name	Species	Firm	Number	Dilution ratio
Alpha Actin Polyclonal antibody	Rabbit	Proteintech	23660-1-AP	1:2000
MyHC	Mouse	Sigma-Aldrich	M4276	1:1000-1:90000
MyoD	Mouse	SantaCruz	sc-377186	1:200-1:1000
MyoG	Mouse	SantaCruz	sc-52903	1:100-1:1000
MuRF-1	Mouse	SantaCruz	sc-398608	1:200-1:1000
PINK1	Mouse	SantaCruz	sc-517353	1:100-1:1000
Parkin	Mouse	SantaCruz	sc-32282	1:100-1:1000
BNIP3	Mouse	SantaCruz	sc-56167	1:100-1:1000
LC3	Rabbit	CST	#12741	1:2000
OPA1	Mouse	SantaCruz	sc-32282	1:100-1:1000
MFN2	Mouse	SantaCruz	sc-100560	1:100-1:1000
DRP1	Rabbit	Abcam	ab184247	1:1000
p-DRP1	Rabbit	Invitrogen	PA5-64821	1:1000
PGC-1α	Mouse	SantaCruz	sc-518025	1:100-1:1000
UCP-1	Rabbit	Sigma-Aldrich	U6382	1:1000
Bax	Rabbit	BOSTER	A00183	1:500-1:2000
Bcl-2	Rabbit	Wanlei	WL01556	1:500-1:1000
Beclin1	Rabbit	CST	#3495	1:2000
VDAC	Rabbit	CST	#4661	1:1000

2. Supplementary Figures S1 to S2

2.1 Effects of different times and different concentrations on myotube differentiation

Myoblasts were stained with Caumas Brilliant Blue staining and photographed under the microscope after treatment of myoblasts without or with MRJPs at concentrations of 0.5, 1.0, 1.5, 2.0, and 4.0mg/mL for 48, 96, and 144 h, respectively, and the experiments showed that 4.0mg/mL MRJPs differentiated myotubes for 144 h with the best results **(Figure S1)**.

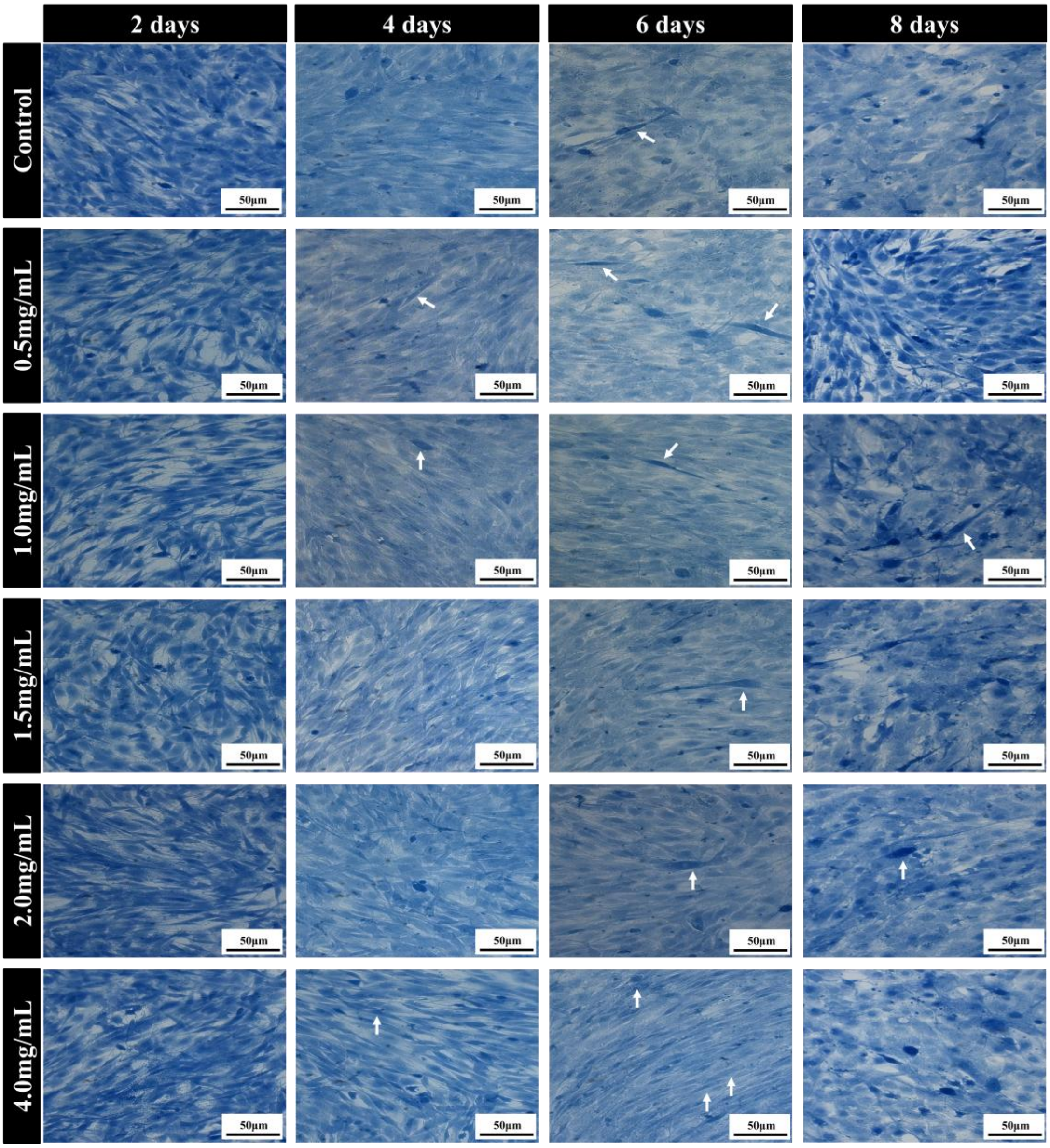

Figure S1: 2, 4, 6 and 8days MRJPs with different intervention concentrations of Caumas Brilliant Blue staining. The white arrows indicate the representative muscular tubes formed by cell fusion.

2.2 Effect of optimal intervention concentration of MRJPs on C2C12 myotube differentiation through the mitophagy pathway

Based on the above experimental results, we selected MRJPs at a concentration of 8.0mg/mL to intervene in C2C12 myotubes to further explore the underlying mechanisms. We extracted mitochondrial proteins in myotubes for protein blotting, and interestingly, the results showed that PINK1 and OPA1 protein expression were increased after MRJPs intervention compared with the control group **(Figure S2 B, and H P < 0.05)**, and there was no significant change in Parkin, BNIP3, DRP1 and MFN2 protein expression **(Figure S2 C, D, F and G)**.

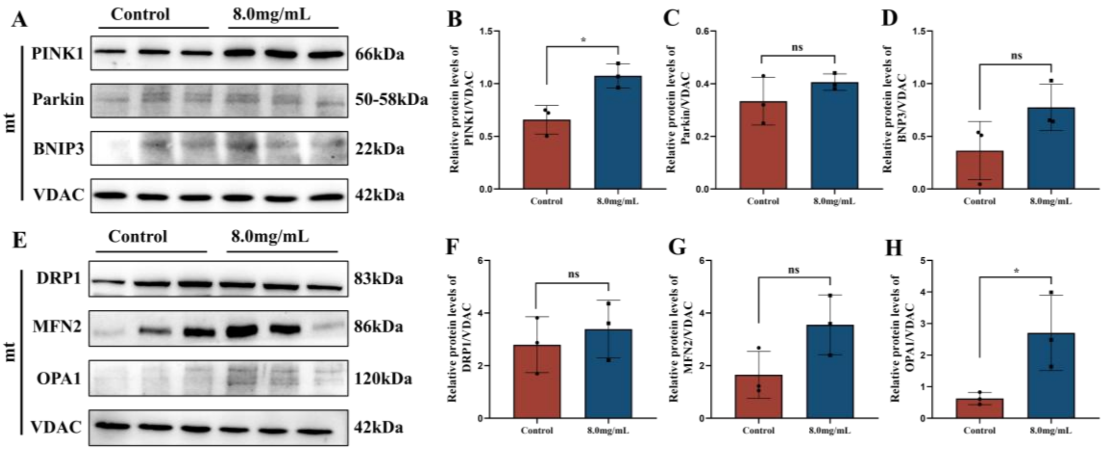

Figure S2: The optimal concentration group of MRJPs improved C2C12 myotube differentiation through the mitophagy pathway. (A-H) Western blotting and quantification of PINK1, Parkin, BNIP3, DRP1, MFN2 and OPA1 in mitochondria. All expressions were normalized to α-actin. $^*P < 0.05$
